# The Potential Role of Vitamin E and the Mechanism in the Prevention and Treatment of Inflammatory Bowel Disease

**DOI:** 10.3390/foods13060898

**Published:** 2024-03-15

**Authors:** Qi Wu, Yi Luo, Han Lu, Tiantian Xie, Zuomin Hu, Zhongxing Chu, Feijun Luo

**Affiliations:** 1 Hunan Key Laboratory of Grain-Oil Deep Process and Quality Control, Hunan Key Laboratory of Forestry Edible Resources Safety and Processing, Central South University of Forestry and Technology, Changsha 410004, China; 20211100421@csuft.edu.cn (Q.W.); 20211100423@csuft.edu.cn (H.L.); 20211100419@csuft.edu.cn (T.X.); 20220100081@csuft.edu.cn (Z.H.); 20221100445@csuft.edu.cn (Z.C.); 2 Department of Gastroenterology, Xiangya Hospital, Central South University, Changsha 410008, China; luoyixy@csu.edu.cn

**Keywords:** vitamin E, inflammatory bowel disease, oxidative stress, anti-inflammation, intestinal barrier, gut microbiota

## Abstract

Inflammatory bowel disease (IBD) includes ulcerative colitis and Crohn’s disease, and it is a multifactorial disease of the intestinal mucosa. Oxidative stress damage and inflammation are major risk factors for IBD. Vitamin E has powerful antioxidant and anti-inflammatory effects. Our previous work and other investigations have shown that vitamin E has a positive effect on the prevention and treatment of IBD. In this paper, the source and structure of vitamin E and the potential mechanism of vitamin E’s role in IBD were summarized, and we also analyzed the status of vitamin E deficiency in patients with IBD and the effect of vitamin E supplementation on IBD. The potential mechanisms by which vitamin E plays a role in the prevention and treatment of IBD include improvement of oxidative damage, enhancement of immunity, maintenance of intestinal barrier integrity, and suppression of inflammatory cytokines, modulating the gut microbiota and other relevant factors. The review will improve our understanding of the complex mechanism by which vitamin E inhibits IBD, and it also provides references for doctors in clinical practice and researchers in this field.

## 1. Introduction

Inflammatory bowel disease (IBD) includes ulcerative colitis (UC) and Crohn’s disease (CD), which are a group of chronic inflammatory diseases that affect the gastrointestinal tract [1]. The incidence of IBD is increasing in various countries [2]. Both UC and CD can cause digestive system disorders and intestinal mucosa inflammation, but the lesion sites of UC and CD are different. UC lesions are generally located in the sigmoid colon and the rectum mucosa and submucosa and even extend to the entire colon. CD usually occurs in all layers of the intestinal wall and in any part of the intestinal mucosa [3]. People with IBD often have symptoms such as rectal bleeding, diarrhea, abdominal pain, and weight loss [4]. So far, there is no definite cause of IBD. Studies have shown that IBD may be caused by interactions between diet, the environment, the immune system, the gut microbiota, and genetic predisposition [5,6]. In severe cases, IBD can even develop into life-threatening colon cancer. Many first-line drugs, such as 5-ASA, sulfasalazine, prednisolone, and immunosuppressants, are commonly used to treat IBD [7]. However, the long-term use of these drugs produces adverse reactions and side effects, such as osteoporosis and damage to liver and kidney functions [8]. To overcome the limitations of current treatment methods, it is necessary to find a safer and more effective treatment for patients with IBD. In recent years, many natural products (NPs) have been found to have obvious anti-inflammatory effects and can effectively relieve IBD, such as flavonoids, polyphenols, quinones, and alkaloids [9,10,11,12]. These NPs have good safety and great potential in the prevention and treatment of IBD.

Vitamin E is an essential fat-soluble nutrient for maintaining life and can be found in various foods from diverse sources [13]. Our prior research indicates that vitamin E possesses potent anti-inflammatory properties and has the ability to inhibit colitis [14,15]. Animal experiments show that vitamin E has potential in the treatment of IBD [16,17,18]. In this paper, we summarized the basic properties of vitamin E, the relationship between vitamin E and gut microbiota, and potential mechanisms in the prevention and treatment of IBD. By elucidating these aspects, this study aims to enhance the understanding of vitamin E’s impact on IBD and offer novel perspectives for the prevention and treatment of IBD.

## 2. Vitamin E

### 2.1. Structure and Physicochemical Properties of Vitamin E

**Figure 1:**
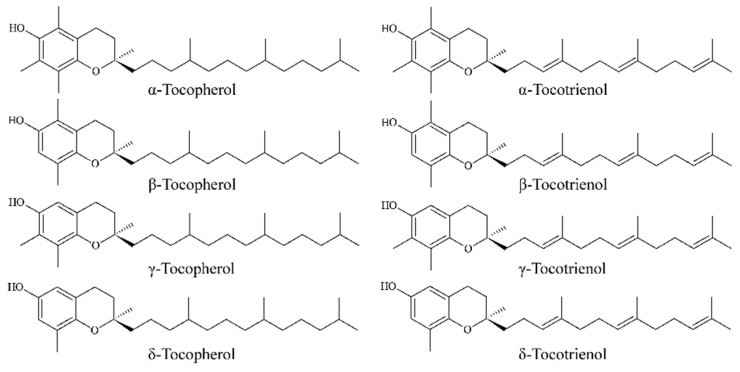
Chemical structures of vitamin E isoforms.

Natural vitamin E belongs to a family of fat-soluble vitamins and includes tocopherols and tocotrienols (Figure 1) [19]. Generally, vitamin E is made up of a chromanol ring and a 16-carbon phytyl side chain. Tocopherols have a saturated side chain, while tocotrienols have unsaturated double bonds in the side chain; this difference may make tocotrienols more hydrophobic in lipid bilayers [20]. According to the number and location of methyl groups on the benzodihydropyran ring, tocopherols and tocotrienols can be further divided into four different isomers, namely α, β, γ, and δ. The more methyl groups in vitamin E isomers, the stronger their effect [21]. The three-dimensional structure of vitamin E also has an effect on its activity, among which the activity of α-tocopherol is the strongest [20]. The benzene ring of vitamin E has a hydroxyl group paired with a heterocyclic oxide atom, which is an active group that is sensitive to oxidants and protects the hydroxyl groups of other substances from oxidative damage and the double bonds in unsaturated fat from oxidative damage [22,23].

### 2.2. Sources of Vitamin E

The sources of natural vitamin E are extensive, but there are differences in the high concentration of vitamin E isomers in different foods. Plant seeds (including common nuts) are rich sources of α-tocopherol and γ-tocopherol [24]. For example, α-tocopherol is mainly found in hazelnuts (>80% of total vitamin E content), almonds (>80% of total vitamin E content), peanuts, and sunflower seeds [25,26,27,28]. γ-Tocopherol is the main form of vitamin E in walnuts (>90% of total vitamin E content), pistachios (>90% of total vitamin E content), pecans, and sesame seeds [24,26,27,28]. Vegetable oil is the main way for human beings to obtain vitamin E daily. Oils of the above-mentioned nuts are also rich in α-tocopherol and γ-tocopherol. Among common edible oils, γ-tocopherol is the most prominent vitamin E in soybean oil (>66% of total vitamin E content) and linseed oil (>97% of total vitamin E content) [26]. α-Tocopherol is more abundant in rapeseed oil (>93% of total vitamin E content), safflower oil (>91% of total vitamin E content), and wheat germ oil (>66% of total vitamin E content) [26]. Another study shows that wild mushrooms are also a good source of α-tocopherol and γ-tocopherol [29]. In addition, α-tocopherol also exists in some fruits and vegetables [30,31]. β-Tocopherol mainly comes from oreganos and poppy seeds [32]. δ-Tocopherol mainly comes from raspberries and edamames [32,33].

**Figure 2:**
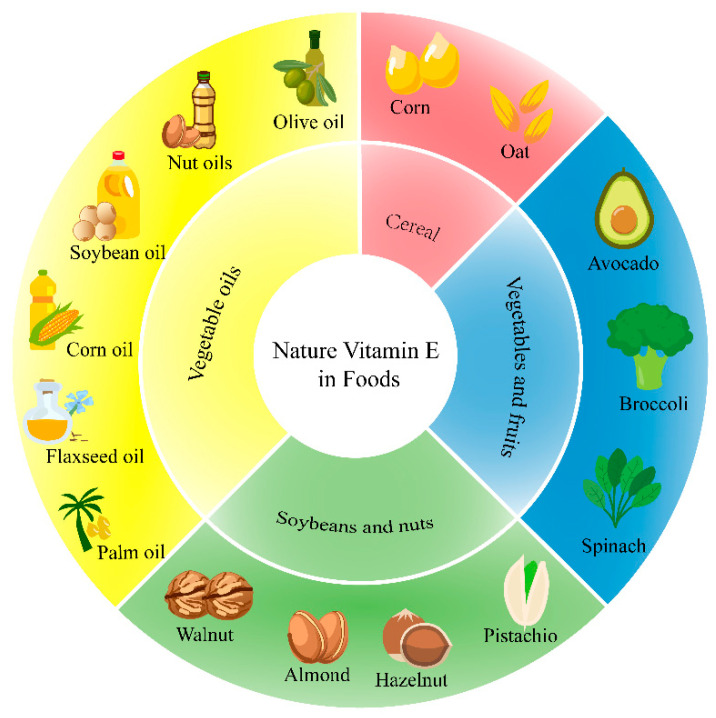
The sources of natural vitamin E in foods.

The main sources of tocopherols are soybean oil, corn oil, olive oil, canola oil, flaxseed oil, walnut oil, and other nut oils. The content of tocotrienols in nuts is much lower than that of tocopherols, which are mainly derived from palm oil and rice bran oil and from grains, such as wheat germ, oats, rice, and corn (Figure 2) [34].

### 2.3. Vitamin E Intake: Recommendations

The Food and Nutrition Committee of the Medical Research Institute (2000) indicated that the estimated average requirement (EAR) for α-tocopherol is 12 mg (27.9 μmol), with a recommended dietary allowance (RDA) of 14 mg/d for individuals under 12 years old [35]. The adequate intake for infants (0–6 months) is estimated at 4 mg, while children aged 7–12 months are recommended to consume 5 mg [35]. RDA dosages are 6 mg/d, 7 mg/d, and 11 mg/d in children aged 1~3 years, 4~8 years, and 9~13 years, respectively [35]. Vitamin E needs increase with age in infants and children but decrease in the elderly regardless of gender [35]. The recommended intake of vitamin E should align with the levels of polyunsaturated fatty acids in food; specifically, 1 g of diene fatty acid or its equivalent necessitates an intake of 0.5 mg of RRR-α-tocopherol [35]. These recommendations aim to prevent symptomatic issues like peripheral neuropathy rather than focusing on health promotion or disease prevention. Due to potential adverse effects, such as increased bleeding tendency, the maximum tolerable intake level for adults is capped at 1000 mg/d of α-tocopherol [35].

Although some studies suggest that high-dose vitamin E supplementation may increase all-cause mortality, more studies show that vitamin E supplementation is safe [36,37]. Berry reanalyzed the evidence with a Bayesian stratified average model and questioned the increased risk of death caused by vitamin E supplementation [38]. The conclusion is that vitamin E supplementation is unlikely to affect the mortality rate regardless of the dose [38]. Therefore, vitamin E supplements are also likely to be ineffective in reducing mortality. It is important to note that if patients are smokers or are taking Warfarin, they should be careful with vitamin E because of the risk of bleeding [38].

## 3. Vitamin E and Inflammatory Bowel Disease

### 3.1. In Vivo

#### 3.1.1. Vitamin E and Oxidative Stress

Oxidative stress is considered an important event in the pathogenesis of IBD and is closely related to the development and clinical symptoms of IBD [39,40]. The overall oxidative capacity (d-ROMs) test and the biological antioxidant potential (BAP) test are used to examine the overall oxidative capacity and antioxidant potential in IBD patients, respectively. A clinical report examined the overall oxidant ability and antioxidant potential in IBD patients by means of the overall oxidant ability (d-ROMs) test and the biological antioxidant potential (BAP) test, respectively. The researchers found that d-ROM values were much higher than normal (250–300 Carratelli units) and were increased over time; BAP values were much lower than normal (2000 μM) and were decreased sharply over time [41]. These results suggest that IBD patients are always in a state of oxidative stress or hyperoxidative stress [41]. In another clinical study, D’Odorico measured antioxidant levels in 83 IBD patients (46 UC and 37 CD patients) and 386 controls. Compared to the control group, they found that the antioxidant concentrations in BD patients were decreased significantly (*p* < 0.0001), especially in patients with active disease [42]. Indian investigators also found that UC patients have higher levels of lipid peroxidation, superoxide dismutase (SOD), catalase, and glutathione (GSH) compared to healthy people and that UC patients have stronger oxidative stress [43]. The study also suggested that the increasing level of oxidative stress may be the cause of the re-emergence of active infection in these patients [43]. These results suggest that the inhibition of lipid peroxidation or the scavenging of oxygen-free radicals may provide a valuable preventive and therapeutic strategy for IBD.

Vitamin E as a powerful antioxidant has the ability to scavenge free radicals and relieve oxidative stress [44]. Clinical trials have shown that vitamin E can reduce levels of oxidative stress markers, such as malondialdehyde (MDA) [45,46,47,48]. However, most of these clinical studies are based on studies of diseases such as diabetes and NAFLD. At present, there is no clinical report on the levels of oxidative stress and the concentrations of different vitamin E isomers in human inflammatory bowel disease after the intake of vitamin E and the effect of these isomers on oxidative stress markers. This research is more about the effect of vitamin E on the markers of oxidative stress in a colitis animal model. Ancha compared the effects of ascorbic acid, phenyl butylnitrone, and α-tocopherol (50 mg/kg) combined with 5-ASA on 2,4,6-trinitrobenzenesulfonic acid (TNBS)-induced colitis in rats [49]. The results showed that the myeloperoxidase (MPO) activity of colon tissue in the 5-ASA treatment group decreased by 49% compared to the TNBS group, and MPO activity decreased by 80% with the addition of α-tocopherol [49]. This showed that α-tocopherol, as an antioxidant, combined with 5-ASA can significantly alleviate the oxidative stress induced by TNBS [49]. In an experimental model of mouse UC induced by DSS, the MDA level in the colon tissue of the DSS group increased by three times compared with that of the blank control group; the tocotrienol-rich fraction (150 mg/kg/d) significantly improved the increase in the MDA level induced by DSS [50]. The experimental results showed that tocotrienols can significantly reduce the oxidative stress induced by DSS [50]. In acetic-acid-induced rat colitis, the glutathione and superoxide dismutase contents in the colonic tissue of mice in the protected group, receiving an intraperitoneal injection of 30 U/kg/d of vitamin E, were significantly lower than those in the colonic tissue of the injured group [17]. These results show that vitamin E may be able to alleviate IBD by reducing oxidative stress. However, due to the complexity of the pathophysiology of IBD, many potential factors must be controlled to obtain clear evidence of the influence of vitamin E therapy on IBD. But there is no doubt that there is a close relationship between oxidative stress and IBD, and antioxidants may be a potential therapeutic strategy.

#### 3.1.2. Vitamin E and the Intestinal Epithelial Barrier

Intestinal barrier dysfunction leads to increased intestinal permeability, susceptibility to bacterial infection, and activation of the immune system [51]. Excessive infiltration of immune cells into the lamina propria and submucosa also leads to cryptinitis and crypt abscess, resulting in epithelial degeneration and necrosis, leading to intestinal dysfunction [52]. Intestinal permeability is regulated by tight-junction proteins [53]. Tight-junction proteins are mainly composed of the transmembrane protein family occludin, claudins, and the peripheral membrane protein family zonula occludens [54]. In DSS-caused colitis, vitamin E regulates the expression of occludin and alleviates the symptoms of colitis [55]. Occludin plays a key role in maintaining tight-junction and intestinal barriers, and the loss of occludin results in increased macromolecular permeability [55]. Vitamin E supplementation (200 IU/kg) can increase the mRNA levels of occludin and ZO-1 by 12–16% [56]. These findings suggest that vitamin E maintains intestinal epithelial barrier integrity by upregulating tight-junction (TJ) protein expression. Vitamin E also has a protective effect on the mucus layer of the intestine [57,58]. Mucoprotein is a major part of the mucin layer, which helps maintain intestinal barrier function and nutrient absorption. Mucoprotein, especially mucoprotein 2 (MUC2), plays a key role in maintaining the thickness of the intestinal mucus layer. Compared to the control group, the RNA levels of MUC2 and type IV collagen alpha 1 chain (COL4A1) were increased after 3 weeks of vitamin E supplementation (160 mg/kg) [58]. COL4A1 is the main component required for the stability of the basement membrane of intestinal epithelial cells. Overall, these findings suggest that vitamin E plays a significant role in preserving intestinal epithelial barrier integrity by upregulating TJ protein expression and maintaining the mucus layer, thereby contributing to the overall function and health of the gut. Interestingly, vitamin E supplementation effectively alleviates colonic fibrosis by inhibiting the expression of TGF-β1-induced fibrosis markers, p-ERK, p-Smad2, and p-JNK, and hinders colonic thickening and shortening in DSS-caused IBD models [59,60].

The presence of excess reactive oxygen species (ROS) in intestinal tissues may further damage intestinal epithelial cells, leading to a compromised intestinal barrier [61]. In a heat-stress-caused intestinal barrier damage model in pigs, vitamin E (200 IU/kg) could attenuate heat-stress-induced oxidative stress damage in the intestine, thereby protecting the integrity of the intestinal barrier [62]. Other investigations have also shown that vitamin E can maintain the integrity and proper functioning of the intestinal barrier by improving oxidative stress in the gut [63,64,65]. In addition, secretory immunoglobulin A (S-IgA) is the main executor of mucosal humoral immunity. S-IgA can protect the mucosal surface from pathogen invasion by inhibiting oxidative stress [66]. Vitamin E supplementation (250 mg/kg body weight (BW)) can increase the S-IgA level in rats with hypoxic intestinal barrier injury [64]. The existing results suggest that vitamin E may reduce intestinal inflammation and even prevent intestinal fibrosis to alleviate IBD by regulating the mucus layer and strengthening the integrity of the intestinal barrier. This provides potential advantages for improving the pathological progress of inflammatory bowel disease.

#### 3.1.3. Vitamin E and the Gut Microbiota

There are potential links between the gut microbiota and the forms of vitamin E and its metabolites. Li and colleagues found that in adults with cystic fibrosis, vitamin E intake is significantly positively associated with *Firmicutes* and their subgroups (e.g., *Tissierellaceae*) and negatively associated with *Bacteroides* [67]. A positive association between vitamin E and *Firmicutes* was also found in an investigation of lactating women in the United States [68]. In a joint investigation of pregnant women, Mandal and colleagues found that an increase in vitamin E intake is related with a decrease in *Proteobacteria*, which can harbor a variety of pathogens and has proinflammatory properties. Meanwhile, vitamin E also synergizes with some micronutrients [69]. Dietary vitamin E and selenium supplements can increase the abundance of *Lariaceae FE2018* and *Ruminococcus NK4A214* in the gut microbiota, which produce butyric acid by fermenting polysaccharides and degrading arabinoxylan and non-starch polysaccharides [70]. Vitamin E supplementation can promote a more favorable gut microbiota by increasing butyrate-producing microorganisms, such as *Roseburia* [71]. These results suggest that vitamin E plays an anti-inflammatory effect by promoting the production of butyric acid.

In general, the intestinal microbiome composition fluctuates more in patients with IBD than in healthy individuals. The fecal microflora of patients with IBD is significantly lower in *Bacteroidetes* and *Firmicutes*, while the proportion of the Proteobacteria phylum is significantly higher [72]. In a DSS-caused-colitis model, the gut microbiota of γ-tocopherol-supplemented mice was positively correlated with control animals but negatively correlated with that of DSS-treated mice [55]. The gut microbiota of the α-tocopherol-supplemented mice was also negatively correlated with that of DSS control mice, although the effect of α-tocopherol appeared to be weaker than that of γ-tocopherol. γ-Tocopherol ameliorates colitis associated with a decrease in rosemary, which is the metabolic product of butyrate. Butyrate is the preferred energy source of intestinal cells and can enhance intestinal epithelial regeneration, which improves barrier function of the intestinal mucosa [55]. In patients with IBD, the butyrate content is negatively correlated with the genetic risk score of IBD [73]. γ-Tocopherol can also change the β-diversity of the gut microbiota and can reduce *Roseburia* depletion in DSS-caused colitis [55]. These results suggest that γ-tocopherol and α-tocopherol can promote beneficial changes in the gut microbiota. In a recent investigation, δ-tocotrienol and its metabolite δ-tocotrienols-13′-carboxychromanol could cause significant changes in the β-diversity. These compounds could change the gut microbiota at the family, genus, and species levels, for example, they could increase the relative abundance of the gut-friendly *Lactococcus* and *Bacteroides*. The investigation also found that high levels of *Lactococcus lactis* are associated with high levels of tocotrienol and its hydrogenated metabolites in the feces [74]. In short, vitamin E affects the production of metabolites by changing the abundance of the gut microbiota.

However, Choi and colleagues found that the intake of a low dosage of vitamin E (0.06 mg/20 g of BW per day) causes more obvious changes in the gut microbiota than a high dosage of vitamin E (dosage: 0.18 mg/20 g of BW per day) [75]. There were significant changes in the abundance of *Proteobacteria* and a decrease in *Verrucomicrobia* compared to the control mice regardless of the dosage of vitamin E. This evidence does not negate the fact that vitamin E alters the gut microbiota. An investigation of the relationship between long-term dietary patterns and the gut microbiota in healthy adults showed no significant relationship between vitamin E intake and the non-disease gut microbiota [76]. These findings highlight the complexity of the gut microbiota and the various factors that affect its composition. Although vitamin E may have some effects on the gut microbiota, the exact mechanism and consistent correlation have not yet been determined. We need further research to understand the relationship between vitamin E intake and the gut microbiota and determine whether there are any significant and repeatable effects.

### 3.2. In Vitro

Vitamin E is present on the membranes of all cells and is particularly abundant in immune cells [77]. The integrity of the immune cell membrane is key to the function of immune cells. The mutual recognition and signal transduction between immune cells or between other cells highly depend on the composition and structure of the cell membrane [77]. Polyunsaturated fatty acids are abundant in the immune cell membrane and are susceptible to oxidative damage due to their high metabolic activity and prophylactic effect against pathogens. Vitamin E is a strong antioxidant that partially protects unsaturated fat from oxidation, maintaining membrane integrity, and protects immune cell structures from oxidative stress [78]. Studies have shown that oxidative stress promotes the infiltration and activation of inflammatory cells, including T cells, macrophages, and neutrophils [79]. However, vitamin E may be able to have an inhibitory effect on the release of inflammatory mediators. Prostaglandin E_2_ (PGE_2_) is an important proinflammatory mediator, and its synthesis rate is mainly controlled by cyclooxygenase (COX)-1 and COX-2 [80]. COX-1 is responsible for maintaining the normal level of prostaglandin in the body, but COX-2 can be activated by the proinflammatory mediator, resulting in excessive synthesis of PGE_2_ at the injured site, thus causing a more serious inflammatory injury [81]. Investigations have shown that γ-tocopherol and its main metabolite γ-CEHC can inhibit PGE_2_ synthesis by macrophages, and this effect is achieved by decreasing COX-2 activity [82]. In addition to inhibiting prostaglandins, vitamin E supplementation reduces the production of proinflammatory cytokines, such as interleukin (IL)-1β, IL-6, and tumor necrosis factor α (TNF-α) [83,84].

Usually, both IL-2 and IL-17 maintain a balanced state, but the levels of IL-2 and IL-17 in patients with IBD are increased [85]. IL-2 is known for its capacity to promote lymphocyte activation and proliferation [86]. IL-2 plays a critical role in regulating the repair processes of intestinal mucosal cells. IL-2 controls intestinal epithelial cell proliferation and cell death by regulating the p52-SHCA and JAK3 signaling pathways, which are critical for maintaining mucosal homeostasis after injury repair [87]. IL-17 plays multiple roles in IBD, including promoting inflammation, affecting intestinal epithelial cells, activating other immune cells, and impairing the integrity of the intestinal mucosal barrier, which affect the development and severity of the disease [88]. Elevated levels of IL-17 have been detected in patients with autoimmune diseases, such as IBD [89]. Vitamin E can inhibit the production of IL-2 and Il-17, as well as the production of proinflammatory chemokines IL-8 and Rantes, and IL-8 plays an important role in the pathogenesis of UC [89]. Experimental studies have shown that IL-8 levels are positively correlated with the degree of inflammation in the colonic mucosa [90]. Vitamin E (200 μg/mL) is found to significantly reduce the production of proinflammatory factors *in vitro*, which may be due to increased cyclic adenosine monophosphate [89]. The pivotal involvement of IL-2 and IL-17 in the pathogenesis of inflammatory bowel disease highlights the potential for modulating their levels as a novel therapeutic approach. Furthermore, given its antioxidant properties, vitamin E shows promise in regulating inflammatory responses, suggesting a prospective role in the treatment of inflammatory bowel disease.

The integrity of the cell membrane may lead to a change in signal transduction and finally lead to a change in function. Some investigations indicate that vitamin E can regulate immune activity by mediating age-related T cell functions, such as naive T cells. The functions of T cells are also inhibited in the presence of ROS [91]. Vitamin E could promote the proliferation of primitive T cells and increased intracellular IL-2 protein levels in aged mice [91]. Immune synapses are formed at the point of contact between T cells and antigen-presenting cells and participate in T cell activation [92]. The zeta chain of the T-cell-receptor-related protein kinase 70, linker for activation of T cells (LAT), phospholipase-c γ, and Vav protein are key molecules involved in the formation of immune synapses [93]. The T cells of elderly mice (500 parts per million (ppm) of vitamin E) indicated significantly higher immune synapses and were 45% higher than mice fed basic levels of vitamin E [94]. Vitamin E could promote the phosphorylation of LAT, especially in the T cells of aged mice [95]. Vitamin E also regulates the Th1/Th2 balance. Under the stimulation of different inflammatory mediators, CD4 helper T cells can differentiate into Th1 and Th2 cells. Th1 cells can promote cytokine production that enhances cell-mediated immune responses. Interferon-γ (INF-γ) is a hallmark cytokine of Th1 immune responses, and IL-4 plays an important role in Th2 cell development. Vitamin E can significantly increase the level of INF-γ in peripheral blood cells, and the immune response of δ-tocotrienol is stronger than that of α-tocopherol [96]. Vitamin E given to allergic donors (12.5–50 μM) reduces IL-4 protein production in human peripheral blood T cells [97]. In patients with colorectal cancer, short-term supplementation with high-dosage vitamin E for 2 weeks can increase Th1 responses and promote T cells to produce IFN-γ [98]. Vitamin E also reduces the production and release of inflammatory mediators in mast cells. Vitamin E can reduce mast cell degranulation by scavenging free radicals, suggesting that vitamin E may have a beneficial effect on inflammation and allergic disease [99,100,101]. Compared with vitamin E deficiency, *in vitro*, vitamin E supplementation can effectively restore the antioxidant status of patients with colorectal cancer and significantly enhance the lytic activity of natural killer cells [102]. In addition, *in vitro*, investigations have shown that vitamin E can enhance the expression of klotho and inhibit the transcript activation of nuclear factor κB (NF-κB), thereby inhibiting the maturation of dendritic cells [103]. Klotho is a kind of anti-aging protein and can protect cells from damage [104]. The abnormal activity of the immune system is closely related to the development of IBD. Given vitamin E’s capacity to modulate immune functions, it holds promise for potential applications in the treatment of inflammatory bowel disease. However, further clinical studies are needed to confirm its safety and effectiveness. Vitamin E may play an immunomodulatory role by regulating the activity of immune cells and inhibiting the release of inflammatory mediators. This regulation may involve effects on inflammatory cytokines and oxygen-free radicals. In *vitro* studies provide a preliminary understanding of cellular and molecular levels, but in *vivo* studies can better reflect the real biological environment. The complex physiological conditions, metabolic pathways, and interactions of the human body may affect the immunomodulatory effects of vitamin E. Therefore, more in *vivo* studies are needed to verify the exact role of vitamin E in the immune system. Although there is no direct evidence to support a modulatory effect of vitamin E on the immunity of IBD, its role as an antioxidant and its protective effect on cell membranes suggest it is a potential immunity modulator. Vitamin E might reduce inflammation and improve the balance of the immune system and is expected to be an auxiliary drug in the treatment of IBD.

**Figure 3:**
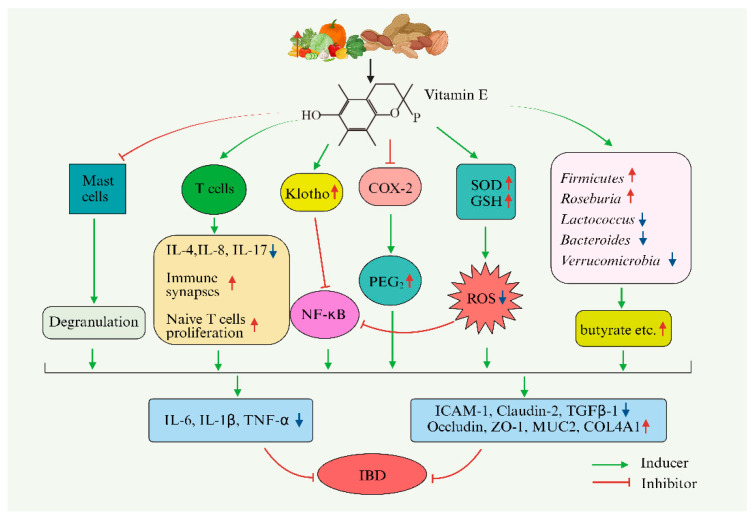
Vitamin E and inflammatory bowel disease. Vitamin E can regulate oxidative stress in the intestine, regulate the immune system, and influence the expression of key inflammation-related genes to regulate the inflammatory response. It maintains the integrity of the intestinal epithelial barrier by regulating the expression of tight-junction proteins, the mucosal immune response, and the thickness of the mucous layer. It also has a certain influence on the composition of the intestinal microbiota. P is the 16-carbon phytyl side chain in the structure of vitamin E. “↑”represents “an increase” and “↓”represents “ a decrease” compared with the level of IBD group.

There is some other evidence regarding the effect of vitamin E on the intestinal barrier. In patients with IBD, increased intercellular adhesion molecule-1 (ICAM-1) protein promotes the recruitment of large numbers of leukocytes to sites of intestinal inflammation, whereas upregulation of claudin-2 expression induces a reduction in and the redistribution of epithelial TJ proteins, which increases the cytokine level and barrier dysfunction [105,106,107]. TNF-α is a cytokine that plays a key role in chronic intestinal inflammation, such as IBD [108]. In HT29 cells, TNF-α can increase intracellular ROS production and promote the expression levels of ICAM-1 and Claudin-2, thereby changing intestinal epithelial barrier function and aggravating inflammation. Supplementation of α-tocotrienol and δ-tocotrienol (50 and 100 μM) could reduce the ROS content in a TNF-α-induced-inflammation model in *vitro* [109]. Based on these findings, the potential mechanism of vitamin E in the prevention and treatment of IBD is summarized in Figure 3.

## 4. Vitamin E Deficiency and IBD

Vitamin E deficiency is a common health problem, especially when the body does not absorb enough fat. Micronutrient deficiencies are not clinically obvious and often require laboratory testing. The current standard common definition of vitamin E deficiency is not clear. But the Institute of Medicine’s Food and Nutrition Committee has shown that the plasma α-tocopherol concentration can be used as an indicator of the vitamin E status and that plasma vitamin E levels are a direct indicator of vitamin E storage in the body. In general, human plasma α-tocopherol levels below 11.6 μmol/L indicate vitamin E deficiency [110]. When the plasma α-tocopherol level is more than 30 μmol/L, it is beneficial to the human body [111]. As the plasma α-tocopherol content varies with the total plasma lipid content, different risk groups may require different definitions. For example, for patients with fat malabsorption, the vitamin E status should be measured using the content of lipid-soluble vitamin E per gram [112,113,114]. The serum α-tocopherol content can also be used to assess vitamin E levels in the body. Vitamin E deficiency is indicated when the serum α-tocopherol content is lower than 8 μmol/L. A serum α-tocopherol content ≥ 30 μmol/L is considered beneficial to human health [115]. Vitamin E deficiency is often known to be associated with IBD, and several observational investigations have shown an inverse association between vitamin E and UC risk [114]. But its pathogenesis is multifactorial. Patients with IBD are often unable to eat normally due to increased abdominal pain, systemic inflammation, hypermetabolism, and adverse drug events. In addition, due to ulcers or loss of the small intestine, the absorption surface area of the intestinal tract is reduced and fat is malabsorbed, which can easily lead to an insufficient supply of fat-soluble micronutrients, especially vitamin E. Vitamin E deficiency can also be caused by excessive intestinal loss, effects of medication, or pure parenteral nutrition without taking proper supplements [116,117].

In a Brazilian investigation of nutritional changes in adolescents with Crohn’s disease, serum micronutrient deficiency rates were found to be much higher in those with CD than in the control group, with vitamin E deficiency being the most prevalent antioxidant vitamin deficiency [118]. This suggests that CD patients may have a higher risk of vitamin E deficiency. Rempel and colleagues reported that in a prospective cohort of 165 patients with IBD, 9% of the patients had low vitamin E levels, including 5% of CD patients and 4% of UC patients [119]. This is similar to an investigation of 97 patients with IBD at Children’s Hospital Boston, of whom 6 (6.2%) were vitamin E deficient. These investigations indicate that both CD and UC patients can experience vitamin E deficiency, although the prevalence may vary. Children and adults with active IBD are at greater risk for vitamin E deficiency than those with inactive colitis. The prevalence of low vitamin E in patients with moderate-to-severe active UC can reach 42.8% [120]. MacMaster et al. also found in their investigation of micronutrient status in patients with quiescent IBD that vitamin E biochemical deficiency is not common in CD and UC patients, and there was no significant difference [121]. Some investigations have also indicated that vitamin E deficiency in patients with IBD is often accompanied by vitamin A deficiency and that general vitamin E levels are significantly and linearly correlated with vitamin A levels [120]. This suggests a potential link between these two vitamins in the context of IBD-related malabsorption or nutritional disturbances.

## 5. Vitamin E Supplementation and Disease Course

Several investigations have examined vitamin E as a therapeutic agent for IBD in animal models (Table 1). Liu and colleagues showed that dietary supplementations of 0.05% α-tocopherol or γ-tocopherol mitigate DSS-caused colon inflammation in mice, inhibit DSS-caused tight-junction protein reduction, and increase circulating lipopolysaccharide-binding protein [55]. Moreover, α-tocopherol and γ-tocopherol differentially regulate the intestinal microbiota in DSS-caused colitis [55]. Similarly, vitamin E (30 and 150 IU/kg) could promote the recovery of DSS-caused ulcerative colitis in rats [122]. Another investigation showed a synergistic effect of vitamin E (100 mg/kg BW) with the antioxidant trace element selenium (0.2 mg/kg BW). This combination was most effective in preventing oxidative damage during the inflammatory response to experimental colitis [18]. Combined administration of vitamin E (30 mg/kg) and selenium (4 ppm/L) significantly reduced the severity of colitis lesions in a rat model of ulcerative colitis induced by trinitrobenzene sulfonic acid [123]. These studies show the potential of vitamin E in the treatment of inflammatory bowel disease in animal models. Vitamin E plays a positive role in relieving intestinal inflammation by inhibiting inflammation, protecting tight-junction proteins, and regulating the intestinal microbiota. Especially, the combined application with antioxidant substances, such as selenium, shows a synergistic effect, which is expected to play a more significant protective role in preventing experimental colitis.

There are few human investigations (Table 2). Elaheh conducted a double-blind, placebo-controlled trial in Toronto to evaluate the effect of antioxidant vitamin E on oxidative stress in patients with CD. The 57 patients were randomly divided into two groups. One group of patients received vitamin E (800 IU) and vitamin C (1000 mg) daily, and the other group of patients received a placebo for 4 weeks. Eighty-one percent of patients had oxidative stress, defined as the amount of pentane in their exhaled breath (6 pmol/kg/min). After 4 weeks, the expiratory pentane content and plasma lipid peroxides were significantly reduced in the vitamin E supplementation group compared to the placebo. But the Chron’s disease activity index and orosomucoid, which are used to assess disease activity, did not change significantly [127]. Mirbagheri and colleagues recruited 14 patients with mild and moderately active UC in an investigation of α-tocopherol enema (8000 IU/d) for 12 weeks. The median Mayo disease activity index (DAI) score before treatment was 8 (range from 4 to 10). Only 3 cases had mild disease activity, and the remaining 11 cases had moderate UC. At week 12, nine patients had mild DAI and five had moderate DAI. Average DAI scores were 8.00 ± 0.48 before treatment, 5.10 ± 0.54 at week 4, and 2.30 ± 0.37 at week 12. The mean erythrocyte sedimentation rate (ESR), a marker of inflammation, decreased significantly after 12 weeks of treatment from 42.26 ± 11.7 to 9.33 mm/h. However, plasma vitamin contents were not significantly different from those before the investigation. High dosages of rectal vitamin E therapy did not cause the feared vitamin E overdose problem. Notably, 12 patients continued to receive vitamin E at the end of the 12 weeks. In the following 5 months, none of the patients who continued treatment relapsed, but the remaining patients had two relapses at weeks 4 and 7 [128]. These investigations suggest that oral or rectal vitamin E supplementation may be beneficial for oxidative stress and disease activity in patients with CD and UC, respectively. The results show that vitamin E has the potential benefits of reducing inflammatory markers and improving disease outcomes. However, it is worth noting that the sample number of these investigations was relatively small, and further research is needed with a larger cohort to confirm and popularize these findings.

## 6. Conclusions

IBD seriously affects public health and disrupts normal daily life. It has been shown that vitamin E plays an important role in reducing oxidative damage, maintaining intestinal homeostasis and mucosal barrier integrity, regulating the inflammatory immune response, and regulating the intestinal microbiota. Vitamin E has a synergistic effect with certain antioxidant micronutrients. All these mechanisms may be related to the occurrence, recurrence, and clinical progression of IBD. Existing experimental and clinical data also show that the most effective approach to alleviate IBD with vitamin E may be involved in the regulation of oxidative stress and inflammatory factor expression.

Notwithstanding the promising data, the literature regarding the role of vitamin E in IBD pathogenesis still exhibits certain limitations. Does vitamin E deficiency lead to a more severe inflammatory disease phenotype or increased inflammatory activity, or is it simply a consequence of disease severity? Recent evidence suggests that vitamin E deficiency is associated with IBD risk and that vitamin E is involved in the environmental–gut microbiota–immune system associated with IBD development. Second, further high-quality investigations are required to assess whether vitamin E supplementation can prevent disease recurrence and whether individuals at high risk should undergo screening for vitamin E deficiency and receive preventive treatment to reduce the risk of IBD. Furthermore, the required circulating levels of vitamin E for IBD prevention and management, along with the optimal alternative dosage and duration, remain to be determined. Nevertheless, the impacts of vitamin E on the inflamed gut and its synergistic effects with micronutrients present a promising area for future research. A deeper comprehension of its functions may pave the way for novel therapeutic strategies. The ongoing and robust collaboration among biochemists, nutritional epidemiologists, laboratory scientists, and clinical researchers will further address numerous unanswered questions and enhance our understanding of the intricate functions of vitamin E, as well as its clinical applications.

## Figures and Tables

**Table 1:** Effect of vitamin E intake on IBD in animal experiments.

Subject	Disease	Intervention	Experimental Period	Result (Compared with the Disease Group)	Ref.
42 Male Sprague–Dawley rats, 9 weeks, 210–230 g	TNBS-induced UC	Basal diet containing 0.025% α-tocopherol	1 week	↓MPO activity, ↓plasma AP, =TBARS levels	[124]
40 Male Spraque–Dawley rats, 200–220 g	TNBS-induced UC	Inject α-tocopheryl acetate, 30 mg/kg	2 weeks	↓MDA, ↓PC, ↓XO	[123]
30 Wistar albino rats, 2–2.5 months, 150–200 g	AA-induced UC	Vitamin E (100 mg/kg) plus Se (0.2 mg/kg)	7 days	↓Scores of macroscopic changes, ↓mean histopathological score, ↓CAT, ↑TAC, ↓OSI, ↓MPO	[18]
Nrf2 (–/–) female C57BL/SV129 mice, C57BL/6J female mice	DSS-induced UC	0.03, 0.1, or 0.3% γ-TmT-enriched AIN93M diets	3 weeks	↓Colon inflammation index, ↓8-oxo-dG	[125]
36 Male BALB/c mice, 5–6 weeks	DSS-induced UC	0.05% α-Tocopherol, 0.05% γ-tocopherol-rich tocopherols	2 weeks	↑Length-to-weight ratio, ↓colitis score, ↓IL-6, ↑occludin, ↓plasma LBP, ↑ZO-1, ↑*Roseburia*, changed β-diversity, ↑*Bacteroides acidifaciens*	[55]
120 Male Wistar rats, 5 weeks, 200 ± 10 g	DSS-induced UC	Oral vitamin E (6, 30, and 150 IU/kg)	2 weeks	↑Body weight, ↑CO score, ↑colon weight/length ratio, ↓degree of colonic injury, ↓IL-6, ↓IL-12, ↓TNF-α, ↓IL-18	[122]
HIFs	IBD	10, 20, 100, 1000 μM TRF	Null	↓HIF proliferation, ↑HIF apoptosis and autophagy, ↓procollagen type I and laminin c-1 production	[126]
36 Male C57BL/6 mice, 6–8 weeks	DSS-induced UC	150 mg/kg/d of TRF, oral	12 weeks	↓DAI, ↓histopathological score, ↑colon length, ↑colon weight, ↓splenomegaly, ↓IL-6, ↓TNF-α, ↓IL-17, ↓MPO, ↓NO, ↓COX-2, ↓p-NF-κB, ↓MDA	[50]

UC: ulcerative colitis; TNBS: 2,4,6-trinitrobenzenesulfonic acid; MPO: myeloperoxidase; AP: alkaline phosphatase; MDA: malonaldehyde; PC: protein carbonyl; XO: xanthine oxidase; DAI: disease activity index; AA: acetic acid; CAT: catalase; TAC: total antioxidant capacity; OSI: oxidative stress index; γ-TmT: a mixture of tocopherols rich in γ-tocopherol; 8-oxo-dG: 8-oxo-deoxyguanosine; LBP: lipopolysaccharide-binding protein; TRF: tocotrienol-rich fraction; HIF: human intestinal fibroblast isolated from CD and UC patients undergoing surgical bowel resection and normal colon segments of patients undergoing resection due to colorectal cancer. “↑”represents “an increase” and “↓”represents “ a decrease” compared with the level of UC or IBD group.

**Table 2:** Interventional studies evaluating the effect of vitamin E supplementation on IBD and clinical course.

Study Design	Number of Patients	Control	Intervention	Follow-Up	Outcomes (Disease Activity Evaluation)	Result	Ref.
Double-blind, placebo-controlled trial	57 Patients with inactive CD	Placebo	Vitamins E (800 IU/kg) and C (1000 mg) daily	4 weeks	↓Breath pentane output	The breath pentane output reduced significantly by vitamin supplementation after 4 weeks compared to the placebo group.	[127]
Open-label study	14 Patients with mild and moderately active UC	Null	α-Tocopherol enema (8000 U/d)	12 weeks	↓Mayo DAI	The mean Mayo DAI score dropped from 8 ± 0.48 to 2.3 ± 0.37 at the end of 12 weeks.	[128]

DAI: disease activity index. “↓”represents “ a decrease” compared with the level of UC or CD group.

## Data Availability

The data used to the paper can be made available by the corresponding author upon request.

## Abbreviations

AA	acetic acid
AP	alkaline phosphatase
BW	body weight
CAT	catalase
CD	Crohn’s disease
COL4A1	type IV collagen alpha 1 chain
COX	cyclooxygenase
DAI	disease activity index
GSH	glutathione
HIF	human intestinal fibroblast
IBD	inflammatory bowel disease
ICAM-1	intercellular adhesion molecule-1
IL	interleukin
INF-γ	interferon-γ
LAT	linker for the activation of T cells
LBP	lipopolysaccharide-binding protein
MDA	Malonaldehyde
MPO	Myeloperoxidase
MUC2	mucoprotein 2
NF-κB	nuclear factor κB
NPs	natural products
OSI	oxidative stress index
8-oxo-dG	8-oxo-deoxyguanosine
PC	protein carbonyl
PGE_2_	prostaglandin E_2_
ppm	parts per million
ROS	reactive oxygen
S-IgA	secretory immunoglobulin A
SOD	superoxide dismutase
TAC	total antioxidant capacity
TJs	tight junctions
TNBS	2,4,6-trinitrobenzenesulfonic acid
TNF	tumor necrosis factor
TRF	tocotrienol-rich fraction
UC	ulcerative colitis
XO	xanthine oxidase
γ-TmT	a mixture of tocopherols rich in γ-tocopherol
